# DOES THE PERCEIVED DOSAGE OF BZRAS DURING TAPERING AFFECT SLEEP QUALITY IN OLDER ADULTS?

**DOI:** 10.1093/geroni/igae098.3952

**Published:** 2024-12-31

**Authors:** Sarah Wong, Michael Mitchell, Karen Camacho, Thien Nguyen, Constance Fung

**Affiliations:** Touro University California, California, United States; Greater Los Angeles VA Health Care System, North Hills, California, United States; Greater Los Angeles VA Health Care System, North Hills, California, United States; University of California, Los Angeles, TORRANCE, California, United States; VA Greater Los Angeles, North Hills, California, United States

## Abstract

Reducing adverse events (e.g., falls) is a primary motivator to deprescribe benzodiazepine receptor agonists (BZRAs) in older adults, but success rates vary despite intensive approaches (e.g., cognitive behavioral therapy for insomnia [CBTI]). Mechanisms such as placebo effects may be unexplored targets for improving deprescribing success. A masked taper is one approach to disrupting these mechanisms. Using data from the masked tapering arm of a 5-year randomized clinical trial that augmented CBTI with a masked taper (clinicaltrials.gov NCT03687086; n=89, 67% male, mean age = 69.2 years, 77.5% White, mean Insomnia Severity Index =13.6), we investigated relationships among BZRA pharmacokinetics (duration of action, receptor localization, lipophilicity), perceived BZRA dosage, and sleep quality. Daily, participants guessed their BZRA dosage taken nightly relative to their baseline dosage (guesses classified as over/concordant/underestimation) and completed diaries assessing sleep quality (sleep efficiency, sleep latency, wake after sleep onset). Mixed models examined the effects of pharmacokinetics on underestimation and perceived dosage (over/concordant/underestimation) on sleep quality. Pharmacokinetic measures were not significantly associated with dosage underestimation (all p-values >.05). Dosage overestimation was associated with improved sleep efficiency, reduced sleep latency and reduced wake after sleep onset) (all p-values <.001). Dosage underestimation was associated with worse sleep quality (all p-values <.001). Pharmacokinetics were not associated with estimation of BZRA dosage in a masked taper, but participants who perceived taking a higher dosage of BZRA reported better sleep quality. Future studies may explore the effects of perceived dosage of other drugs (such as analgesics) to aid in masked tapering efforts.

